# Health in a Digital Age: From Determinants to Dynamic Systems

**DOI:** 10.34133/hds.0465

**Published:** 2026-05-15

**Authors:** Michelle A. Williams

**Affiliations:** Professor of Epidemiology and Population Health, Stanford University School of Medicine, Stanford, CA, USA.

The social determinants of health framework transformed how we understand population health [1]. By demonstrating that social and economic conditions—education, income, housing, and employment—shape health outcomes more powerfully than medical care alone, this body of work fundamentally reoriented public health research and policy. However, these frameworks were developed in an era of periodic surveys, limited data linkage, and minimal digital mediation of daily life. Today, algorithms shape health behaviors and beliefs, platforms mediate access to care, and digital systems increasingly determine who gets seen, what gets measured, and how resources are allocated.

In this issue, Li and colleagues [2] introduce the Health Elements framework—an approach that conceptualizes health as an emergent outcome of interacting biological, behavioral, social, environmental, and technological domains. The framework builds explicitly on the social determinants tradition while extending it in 2 important directions: first, by treating technological elements as a structurally independent domain rather than a neutral tool applied to health; and second, by shifting the central research question from which factors matter to how factors interact, under what conditions, and across which time horizons.

The case for treating technology as a first-order domain is compelling [3,4]. Digital systems now introduce genuinely novel mechanisms of health influence (e.g., algorithmic decision-making, platform-mediated behavioral environments, artificial-intelligence-enabled diagnostics, and real-time data feedback loops) that have no functional analog in predigital frameworks. More importantly, technological elements possess a uniquely cross-domain modifying capacity: They restructure how biological, behavioral, social, and environmental factors operate, interact, and scale. In addition, they occupy a reflexive position in health systems, functioning both as determinants of health and as the infrastructure through which other determinants are measured and acted upon.

A particular strength of this framework is its attention to what technology’s absence reveals. In low- and middle-income settings, limited surveillance capacity, lack of interoperable records, and low digital literacy are themselves important features of the technological environment that shape health outcomes and constrain disease detection [5]. The chronic kidney disease (CKD) of uncertain etiology example in the paper illustrates this very well: Among agricultural workers in Central America and South Asia, the technological domain is defined by gaps that impede both detection and etiological investigation. This framing avoids the trap of treating Health Elements as relevant only where digital infrastructure is abundant.

The authors’ treatment of “emergence” deserves attention. In complexity science, emergence refers to system-level outcomes that arise from interactions among components and cannot be predicted from—or reduced to—the properties of any individual component [6]. This is fundamentally different from additive effects, where multiple factors each contribute separately and the outcome equals the sum of individual contributions. The distinction matters methodologically, as it is important to appreciate that genuine emergence is characterized by nonlinearity, context dependence, and feedback dynamics that standard regression-based approaches cannot capture.

The China CKD example makes this concrete. The rise of diabetes as the leading cause of chronic kidney disease cannot be attributed to biological change alone. It reflects the convergence of urbanization, behavioral transition, environmental exposure, and health system capacity—a pattern no single-domain model could have anticipated. Crucially, this reframing shifts the locus of prevention: Rather than placing the onus solely on individuals to change their behavior, it directs attention to the policies, environments, and social conditions that constrain or enable those behaviors in the first place. This is not merely a call for more variables in our models; it is an argument for different kinds of models—and different kinds of interventions—altogether.

The framework’s methodological implications are substantial. The authors are careful to distinguish Health Elements interaction from conventional statistical interaction terms. System dynamics models, agent-based models, and network-based approaches treat interaction as dynamic structure rather than parametric modification—capturing feedback loops, time delays, and emergent population-level patterns that arise from individual-level processes [7]. These methods complement artificial intelligence approaches while addressing their limitations: Predictive capacity alone is insufficient when the goal is to identify actionable interventions.

Equally important is the attention to temporal dynamics. Different domains operate at characteristically different time scales; for example, biological processes over hours, behaviors over months, social conditions over decades. Cross-temporal interaction, as the authors define it, refers to how slowly changing conditions modify the vulnerability within which faster-changing processes operate. This is analytically distinct from contemporaneous statistical interaction and requires methods that preserve domain-specific temporal resolution [8].

No framework is without limitations. The Health Elements approach demands data integration across domains that remain siloed in most health systems. It requires analytical methods (e.g., system dynamics, agent-based modeling, and hybrid causal-machine-learning approaches) that are not yet standard in epidemiological training. In addition, its added complexity must be justified empirically, particularly in contexts where simpler models may suffice. The authors acknowledge this, insisting that complexity must earn its place through demonstrated gains in prediction, explanation, or equity.

There is also the equity challenge. High-frequency biological and behavioral data (e.g., from wearables, smartphones, and electronic health records) may dominate inference simply because they are more granular, not because they are more causally influential. Meanwhile, distal but powerful social and environmental influences may be underweighted because of coarser measurement. If certain populations remain underrepresented in digital data streams, data-intensive approaches risk reinforcing the very disparities they aim to address [9].

Underlying all of this is the data challenge. The Health Elements framework presumes the availability of high-quality, linkable data across biological, behavioral, social, environmental, and technological domains—a presumption that outpaces current reality in most settings. Data quality remains uneven. For example, clinical data captured for billing purposes differ fundamentally from research-grade measurements; self-reported behavioral data are subject to recall bias and social desirability effects; environmental exposures are often estimated from models rather than measured directly [10]. Completeness is equally problematic: Electronic health records capture encounters, not health; wearables capture those who wear them; administrative datasets reflect administrative categories rather than lived experience. Accessibility remains constrained by proprietary systems, institutional silos, and regulatory fragmentation—even when data exist, researchers often cannot link them. In addition, governance presents perhaps the deepest challenge: As multimodal data integration expands the surface area for privacy violations, reidentification, and misuse, robust frameworks for consent, security, and accountability become essential [11]. The Health Elements framework will only be as strong as the data infrastructure that supports it.

Why does this framework matter now? The COVID-19 pandemic demonstrated with painful clarity that health outcomes arise from interacting systems rather than isolated causal pathways. Biological susceptibility intersected with social vulnerability, occupational exposure, housing conditions, institutional trust, and digital access to information and services [12]. No single-domain model could have predicted—or explained—the stark inequities in transmission, severity, and mortality that emerged.

The UK’s COVID Symptom Study offers an instructive, if imperfect, attempt to operationalize something similar to a Health Elements approach in real time [13,14]. Launched in 2020 March by King’s College London and the health technology company ZOE, the app enrolled over 4 million contributors who reported symptoms daily, enabling near-real-time surveillance of disease spread at the local level. The study integrated self-reported behavioral data (symptoms and activities), biological confirmation (polymerase chain reaction testing of symptomatic users), social and demographic information (age, occupation, and location), and technological infrastructure (app-based data collection and machine learning prediction). It identified anosmia as a key symptom weeks before official recognition, detected regional hotspots before they triggered formal alerts, and tracked vaccine effectiveness in real populations [15]. Yet, the study also revealed the framework’s limitations: Participants skewed young, female, and higher socioeconomic status; older adults and those without smartphones were systematically underrepresented; and the voluntariness of participation introduced selection biases that required careful statistical adjustment [16]. The lesson is instructive: integrated, technology-enabled surveillance can generate actionable insights that no single domain could produce alone, but only if equity and representativeness are built into the design.

As health systems confront multifactorial challenges (e.g., chronic disease, population aging, climate-related risks, and widening inequities), single-domain interventions will prove insufficient. The Health Elements framework offers a promising scientific architecture for studying how these challenges interact and evolve, while keeping equity and governance at the center of the analytic enterprise.

Li and colleagues [2] have provided a thoughtful extension of the social determinants tradition for a digitally mediated world. The individual ideas have precedents (e.g., systems thinking, digital determinants, and life-course epidemiology), but the value lies in the synthesis: a framework that hangs together and points toward concrete methods. Its ultimate worth will depend on empirical performance: whether it generates insights that simpler models cannot and whether it advances health equity rather than merely optimizing prediction. That is the work ahead.
